# Polysomnographic titration of non-invasive ventilation in motor neurone disease (3TLA): study protocol for a randomised controlled trial

**DOI:** 10.1186/s13063-024-08464-4

**Published:** 2025-01-06

**Authors:** David J. Berlowitz, Dominic Rowe, Mark E. Howard, Amanda Piper, Marnie Graco, Sabine Braat, Bhajan Singh, Tanara Viera Souza, Natasha Lannin, Alistair McLean, Abbey Sawyer, Kate A. Carey, Yasmin Ahamed, on behalf of the 3TLA Trial Group, Uwe Aickelin, Vinod Aiyappan, Caroline Chao, Deanne Curtin, Kim Dalziel, Liam Hannan, Anne Holland, Matthew Kiernan, Chris Kosky, Collette Menadue, Chris Michael, Linda Rautela, Bec Sheean, Irene Szollosi, Paul Talman, Gethin Thomas, Ostoja Steve Vucic, John Wheatley, Peter Wu

**Affiliations:** 1 Department of Physiotherapy, Melbourne School of Health Science, University of Melbourne, Melbourne, Australia; 2 Victorian Respiratory Support Service, Austin Health, Melbourne, Australia; 3 Institute for Breathing and Sleep, Melbourne, Australia; 4 Centre for Motor Neurone Disease Research, Macquarie University, Sydney, Australia; 5 Department of Medicine, Medicine, Dentistry and Health Sciences, University of Melbourne, Melbourne, Australia; 6 Department of Respiratory and Sleep Medicine, Royal Prince Alfred Hospital, Sydney, Australia; 7 Camperdown Central Clinical School, Faculty of Medicine, University of Sydney, Sydney, Australia; 8 Centre for Epidemiology and Biostatistics, Melbourne School of Population and Global Health, The University of Melbourne, Melbourne, Australia; 9 MISCH (Methods and Implementation Support for Clinical Health) Research Hub, Faculty of Medicine, Dentistry and Health Sciences, The University of Melbourne, Melbourne, Australia; 10 Department of Pulmonary Physiology and Sleep Medicine, Sir Charles Gairdner Hospital, Perth, Australia; 11 School of Human Sciences, University of Western Australia, Crawley, Australia; 12 West Australian Sleep Disorders Research Institute, Perth, Australia; 13 Allied Health, Alfred Health, Melbourne, Australia; 14 Department of Neuroscience, Central Clinical School, Monash University, Melbourne, Australia

**Keywords:** Motor neurone disease, Non-invasive ventilation, Polysomnography, Sleep study, Amyotrophic lateral sclerosis, Chronic respiratory failure

## Abstract

**Background:**

Non-invasive ventilation (NIV) uses positive pressure to assist people with respiratory muscle weakness or severe respiratory compromise to breathe. Most people use this treatment during sleep when breathing is most susceptible to instability. The benefits of using NIV in motor neurone disease (MND) are well-established. However, uptake and usage are low (~ 19%) and there is no consensus on how to best implement NIV in MND in Australia. Consequently, clinical practice models are highly variable. Our team has recently provided evidence that specific and individualised NIV titration using a sleep study (polysomnography; PSG) leads to better outcomes in people with MND. However, for this clinical practice model to result in sustained benefits, evidence of effectiveness across multiple sites, as well as culture and practice change, must occur.

**Methods:**

A two-arm, assessor-blinded, individual participant randomised controlled trial in MND care centres across Australia will be undertaken. Two-hundred and forty-four participants will be randomised (1:1) to either the intervention group (PSG-assisted commencement of NIV settings; PSG) or a control group (sham PSG). Participants will be asked to use their NIV device for 7 weeks and will then return for follow-up assessments. Respiratory, sleep and patient-reported outcome measures will be collected at baseline and follow-up. The primary aim is to determine if the proportion of participants using NIV for > 4 h/day during the intervention period is higher in the PSG than the control group. A process evaluation, health economic evaluation and 12-month cohort follow-up will be undertaken and reported separately.

**Discussion:**

The results of this trial will demonstrate the effects of PSG-assisted titration of NIV on usage of NIV in people with MND. We hypothesise that the PSG intervention will improve synchrony between the user and the machine, which will lead to greater NIV usage compared to the control group.

**Trial registration:**

ClinicalTrials.gov NCT05136222. Registered on November 25, 2021.

**Supplementary Information:**

The online version contains supplementary material available at 10.1186/s13063-024-08464-4.

## Background

Most people with motor neurone disease (MND)/amyotrophic lateral sclerosis (ALS) survive 2 to 3 years from diagnosis as progressive motor weakness leads to respiratory failure and death. It was estimated in Australia in 2015 that MND was associated with economic and burden of disease costs of $1.13 million per person diagnosed, with a total annual cost of $2.37 billion [1]. These costs are primarily driven by the loss of life due to premature mortality and the immense financial and disability burden to the individual and their families coping with rapid decline and death.

The benefits of non-invasive ventilation (NIV) to treat chronic respiratory failure and reduce the rate of respiratory function decline are well-established in people with MND. A 2006 single-site randomised controlled trial (RCT) of NIV versus no-NIV demonstrated a modest overall survival benefit. Five single-site cohorts have also associated NIV use with increased survival in MND, including a 20-year study conducted by members of the current research team [2–6], which demonstrated that NIV slows respiratory decline and improves survival by a median of 13 months [4].

Despite these benefits [7], methods for commencing NIV are variable, and there is little consensus on how best to implement NIV. A number of clinical practice models using a variety of evaluation tools have been reported, ranging from inpatient hospital assessments to ambulatory or home-based models [4, 7–9]. Some of the variation in practice is attributable to inequitable patient access and local, state and national differences in healthcare funding and models [10, 11].

Undertaking an overnight sleep study (polysomnography; PSG) to support the initiation of NIV uniquely provides direct observation of patient-ventilator interaction, respiratory control and an assessment of sleep quality. Patient-ventilator asynchrony is a mismatch between neural (user) and mechanical (ventilator) drive and timing [12] and has been described in the critical care [13] and NIV literature [14, 15]. Uncontrolled studies have demonstrated frequent patient-ventilator asynchrony in those using nocturnal NIV following daytime clinical titration [14–16], which improved with setting adjustments [15, 16]. Associations have been found between increased levels of patient-ventilator asynchrony and more arousals from sleep [17], less rapid eye movement (REM) sleep, lower sleep efficiency [15], worse nocturnal gas exchange [15] and reduced NIV tolerance [18].

In a recent single-site RCT (*n* = 56 new initiations of NIV for hypercapnic respiratory failure, 36 with MND), patient-ventilator asynchrony event rates were reduced at trial conclusion in those who underwent PSG-assisted setting optimisation; median (25th to 75th percentile) PSG: 26/h (12 to 68) versus control: 41/h (28 to 182), *p* = 0.046 [16]. Mean difference in average daily NIV use (defined as average daily NIV use during the treatment period minus the average daily use during the acclimatisation period) in those initially non-adherent to NIV (average NIV use < 4 h/day) was more than one and a half hours higher in the PSG group versus the control group (mean difference (min) (95% CI) PSG: 95 (29 to 161) vs control: − 23 (− 86 to 39)). A subset analysis of those with an MND diagnosis showed a greater number of participants who were initially non-adherent became adherent after PSG titration compared to control (Hannan, LM unpublished observations) (PSG (*n* = 19): adherent pre = 13, adherent post = 16; control (*n* = 17): adherent pre = 9, adherent post = 9).

This trial confirmed other reports that NIV is effective; it controls hypercapnia equally well despite differing devices and care models [9]. In addition, this trial demonstrated that optimising ventilatory control and synchrony of the ventilator with the patient through the use of full polysomnography increases usage by a clinically important amount. However, these preliminary findings need to be tested in other settings in order to facilitate widespread practice change.

## Methods

### Aims and hypothesis

The primary aim of this RCT is to determine if, in people with MND who are referred for NIV, the proportion of participants using NIV for > 4 h/day during the intervention period is higher in the PSG (intervention) group than the control group. The primary hypothesis is that implementing NIV for people with MND with or at risk of chronic hypercapnic respiratory failure utilising PSG (intervention) to evaluate and titrate settings will lead to higher NIV usage compared to the control group. Usage is defined as using NIV for > 4 h/day while the participant is alive during the intervention period.

The secondary aims of this RCT are (i) to determine if total daily NIV use (hours/day) is greater in the PSG (intervention) group than in the control group; (ii) to evaluate whether the PSG group is superior to the control group in the change from baseline measures of gas exchange, respiratory function and patient-reported outcome measure (PROM) test scores as well as follow-up (review) physiological and objective sleep quality measures; and (iii) to examine differences in carer burden among people who care for participants in the PSG (intervention) and control group. Further exploratory aims are to determine whether particular patient-ventilator asynchrony sub-event types (ineffective efforts, double-trigger, multiple trigger) differ between the PSG and control group (RCT); to determine whether particular patient-ventilator asynchrony sub-type events are associated with objective NIV usage (RCT and 12-month cohort); and to examine associations between objective NIV use, survival and participant PROMs (RCT and 12-month cohort).

### Design

**Fig. 1:**
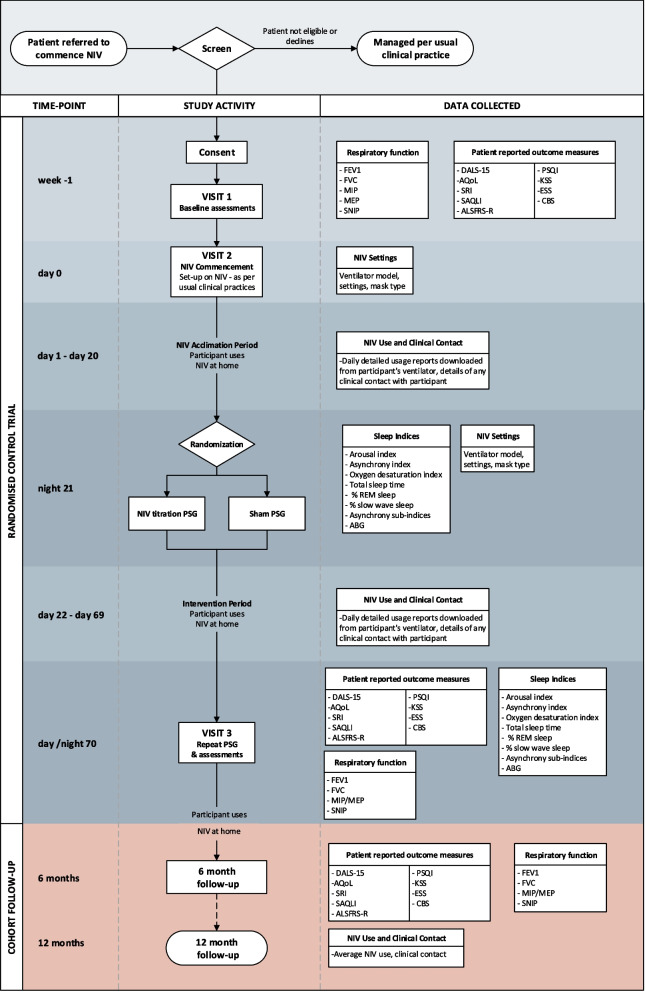
Participant flow through the 3TLA trial. NIV non-invasive ventilation, PSG polysomnography, PROMs patient-reported outcome measures

This trial of PSG-assisted commencement of NIV in MND follows similar methodology of the previous single-site trial [16]. The 3TLA trial is a two-arm, parallel group, superiority multi-centre trial conducted at MND care centres across Australia. These include Austin Health (Melbourne), Flinders Medical Centre (Adelaide), Macquarie University (Sydney), Royal Prince Alfred Hospital (Sydney), Sir Charles Gairdner Hospital (Perth), The Prince Charles Hospital (Brisbane) and Westmead Hospital (Sydney). Additional sites may be added as the trial progresses. The trial has been designed according to SPIRIT guidelines [19], will be conducted in accordance with the principles of Good Clinical Practice and reported according to the CONSORT and the complimentary StaRI standards for implementation studies [20]. Eligible participants will be randomised on a 1:1 basis to either the intervention group (daytime and PSG-assisted commencement of NIV settings; PSG) or control group (daytime commencement of NIV settings and sham PSG). The anticipated flow of participants through the trial is represented in Fig. 1.

### Participants

Consecutive individuals referred for implementation of NIV will be identified. Those who are interested in participating will be provided with a Participant Information and Consent Form by a member of the research team. Participants who meet the following inclusion criteria will be eligible to participate: (i) medically stable, (ii) suitable for outpatient implementation of NIV, (iii) clinical indication to commence long-term NIV according to local protocols, published guidelines [21, 22] and/or specialist opinion, (iv) confirmed clinical diagnosis of MND and (v) age > 18 years. Individuals will be excluded if they are (i) medically unstable, (ii) demonstrate hypoventilation attributable to medications with sedative/respiratory depressant side-effects, (iii) have previously used NIV for > 1 month in the previous 3 months, (iv) are unable to provide informed consent, (v) have experienced previous intolerance of NIV or (vi) are pregnant.

### Procedures

#### Visit 1 (day 0)

##### Baseline data collection

All participants who meet the selection criteria and agree to participate will be invited to undertake visit 1. Baseline data collection will involve collection of written informed consent, screening data, general, respiratory and PROMs (listed below). Clinical measures (i.e. respiratory function) will be undertaken by the usual clinical staff at each site. Non-clinical measures (i.e. general information and PROMs) will be collected with the assistance of research staff. The specific measures collected are listed below.

General:

- Demographic (age, sex, education level) and participant characteristics (height or arm span for non-ambulant [cm], weight [kg], medical history and current MND medications)

Respiratory function (or most recent results if undertaken in the preceding 2 weeks):

- Lung function (spirometry measures of forced expiratory volume in 1 s [FEV_1_], forced vital capacity [FVC])
- Maximal inspiratory/expiratory (MIP/MEP) and sniff nasal inspiratory pressure (SNIP)

PROMs:

- Daytime sleepiness (Karolinska Sleepiness Scale [23] and the Epworth Sleepiness Scale [24])
- Function (Amyotrophic Lateral Sclerosis Functional Rating Scale (Revised)) [25]
- Dyspnoea (Dyspnoea Amyotrophic Lateral Sclerosis) [26]
- Health-related quality of life (Assessment of Quality of Life [AoQL-8D] [27], Severe Respiratory Insufficiency Scale [28] and Calgary Sleep Apnoea Quality of Life Index [29])
- Sleep quality (Pittsburgh Sleep Quality Index) [30]
- Carer burden (Caregiver Burden Scale) [31]

##### Daytime NIV initiation

Participants in both groups will receive a standardised daytime commencement and titration of NIV performed by the same experienced clinical staff (respiratory physiotherapists, scientist, nurses or physicians, depending on site) who would normally undertake this task clinically. All sites in the proposed RCT are experienced providers of NIV in MND, practice according to current guidelines [21, 22, 32] and routinely address patient interface and NIV efficacy.

Daytime NIV commencement will involve individualising masking and NIV settings to subjectively optimise comfort, leak, patient-ventilator synchronisation, minute ventilation and oxygen saturation (SpO_2_). The NIV devices typically used clinically at each site will be utilised for this trial. These devices provide remote access or data extraction ensuring objective recording of adherence (usage). Devices will deliver bi-level NIV with a back-up respiratory rate. Rise time, inspiratory time, trigger and cycle sensitivities will be adjusted by the clinical staff as per usual practice.

#### Acclimatisation period (day 0–day 20)

Following NIV commencement, participants will be sent home with the device and asked to wear it for a 3-week ‘acclimatisation’ period. During this time, the clinical team can adjust settings as deemed clinically necessary. Standard clinical procedures (e.g. phone calls and home visits) will not be restricted and will operate as per the usual process. Non-invasive ventilation usage data (hours/day) will be recorded throughout this period. Participants who cease NIV during the acclimatisation period will have their reasons for cessation recorded.

#### Visit 2—polysomnography and allocation (night 21)

##### Polysomnography

After the 3-week acclimatisation period, all participants will return to the sleep laboratory for a full PSG as per standard procedures at each clinical site. A standard treatment or NIV PSG montage with additional device outputs will be provided to all sites for consistency. Participants will be set up by experienced sleep scientists. Full PSG will include standard electroencephalography, left and right electro-oculography, submental and diaphragm electromyography, measurement of airflow using the NIV device, body position, thoracic and abdominal respiratory effort bands and video recording. Oxygen saturations will be measured continuously with the use of a pulse oximeter and a finger probe. Measures of transcutaneous carbon dioxide (PtcCO_2_) will be performed using transcutaneous capnography with the probe heated to 43 °C. Once set up, an arterial blood gas (ABG) sample will be collected. Upon completion, the PSGs will be staged, scored [33] and reported using published criteria [34].

##### Randomisation

A computer-generated randomisation list will be provided by an unblinded independent statistician, using randomly permuted blocks stratified by centre. The participants’ treatment allocation will be revealed by the sleep scientist or another unblinded member of the research team immediately before PSG commencement at visit 2. The allocation sequence will be concealed using a centralised trial database and will not be revealed to participants of either group or the clinical team. The unblinded individual will not be involved in data analyses or the clinical care of participants during the intervention period.

##### Intervention group

The intervention group will undergo an attended overnight titration PSG. An experienced sleep scientist or registered nurse will adjust the NIV settings (and mask) using clinical experience and site-specific practices. In addition, each site will receive a standardised laboratory manual which they are able to refer to throughout the study period. A summary of instructions provided within the laboratory manual are outlined in Table 1.

**Table 1:** Summary of laboratory manual instructions on the titration of non-invasive ventilation provided to trial sites

General	EPAP	IPAP	Patient-ventilator asynchrony
• Commence EPAP and IPAP 2 cmH_2_O lower than that determined during the daytime trial. • Leave other settings as per the daytime trial. • Carefully observe the interaction between respiratory channels when titrating (chest and abdominal movements, airflow signal, mask pressure, leak). • Before altering settings, look for excessive leak first.	• Increase EPAP in 1 cmH_2_O increments in the presence of obstructive events. • Trial an increase in EPAP if ineffective efforts are observed (in the absence of significant leak). • Maintain the initial IPAP-EPAP difference until obstructive events are controlled.	• Hypoventilation should be identified and primarily addressed through an increase in IPAP (hence pressure support)—a rise in PtcCO_2_ of 10 mmHg above awake, supine, resting PtcCO_2_ should prompt increases in pressure support. • If the transcutaneous CO_2_ signal is thought to be inaccurate, re-apply and review before acting on the result. • An increase in IPAP should be trialled to minimise non-obstructive hypopnoeas, flow limitation or to improve SpO_2_—if signs of partial upper airway obstruction persist, trial a further increase in EPAP while maintaining pressure support.	• If significant patient-ventilator asynchrony is observed, all efforts to reduce leak should be made before altering respiratory rate, trigger/cycle or inspiratory time. Pressure changes should be made before altering other parameters. • Respiratory rate should not be increased more than 4 breaths per minute above the initial setting. • Sleep scientists made changes to ventilator parameters during the titration study in order to rectify problems identified overnight. The attending scientist would typically make small incremental changes every 10 to 20 min once a problem was identified in order to determine the effect. The attending sleep scientist would make further adjustments according to the perceived response. This would include increasing or decreasing pressure levels (both pressure support and EPAP as required) or altering timing criteria at their discretion. These data would be used by the clinicians analysing the study to determine the optimal settings for the individual participant after reviewing all of the polysomnographic data.

*Abbreviations*: *CO*_*2*_ carbon dioxide, *cmH*_*2*_O centimetre of water, *EPAP* expiratory positive airway pressure, *IPAP* inspiratory positive airway pressure, *mmHg* millimetre of mercury, *PtcCO*_*2*_ transcutaneous carbon dioxide, *SpO*_*2*_ oxygen saturation

In the days following, the empirical NIV commencement settings will be revised based on the report recommendations from the PSG, as described under ‘Intervention period (day 22–day 69)’, below.

##### Control group

The control group will undergo an attended overnight PSG without any titration to correct ventilator abnormalities. Masking issues (leak, noise, fit, etc.) identified by the participants in both arms will be addressed. In the control arm, however, potential issues observed during the PSG but not identified by participants will not be addressed [16]. This sham PSG thus controls for the effect of the PSG per se without contamination.

#### Intervention period (day 22–day 69)

Following the PSG, a member of the research team, unblinded to group allocation, will then program the updated ventilator settings onto data cards, SD cards or USB sticks to be sent to participants with instructions on how to update their devices. Devices with a connection to cloud-based monitoring systems, such as AirView and Care Orchestrator, may have their settings updated using this software as appropriate, providing group allocation blinding is maintained.

Following the PSG, participants will enter the intervention period, during which there are no planned reviews or additional interventions. Participants will have access to their clinical teams as per standard procedures at each site. All contacts, interventions, adjustments to settings, changes to interfaces, etc. will be recorded and be included in the health economic analysis (the methodology of this will be reported separately). Non-invasive ventilation usage (hours/day) will be extracted via detailed reports from online platforms (e.g. AirView) or NIV data cards.

#### Visit 3—PSG2 and follow-up assessment (day/night 70)

Following completion of the intervention period, participants will be asked to return to the study site for a follow-up assessment and second PSG; the follow-up assessment will mirror measures collected at baseline. Unattended home PSGs may be offered as an alternative to in-laboratory if clinically indicated. No settings will be adjusted during the PSG at visit 3. The sleep scientist performing analysis of these studies will be unaware of treatment allocation.

#### 6- and 12-month cohort follow-up

Following completion of the intervention period, participants will enter a 12-month open label cohort which will be reported separately from the RCT. Data collection timepoints within the cohort study will be conducted at 6 and at 12 months after the NIV initiation (visit 1). The cohort follow-up data collection will be undertaken within ( ±) a 2-week period of each designated timepoint and will be organised in accordance with usual clinic follow-up to minimise burden to participants and clinical teams. During each visit, survival and PROMs will be collected.

### Data analysis

A stand-alone statistical analysis plan will be finalised for the RCT before unblinding of the study database, detailing all analyses methods. Analyses will include randomised participants grouped according to their randomised intervention group. The primary estimand of clinical interest is the treatment effect of PSG (intervention) compared to control to titrate NIV therapy on usage of NIV (> 4 h/day) during the intervention period or until death (whichever occurs first), regardless of whether participants are prescribed a disease modifying medication, are non-adherent or discontinue NIV, experience adverse events or are admitted to hospital with an acute event during the intervention period. This estimand represents how patients are treated in clinical practice and assumes death is not a failure of the intervention under investigation.

For the primary outcome, we will use log-binomial regression adjusted for the average NIV usage per day during the acclimatisation period (in hours) to estimate the primary estimand, using control as the reference group. Secondary continuous outcomes will be analysed using linear regression adjusted for the baseline value, where applicable, with log transformation applied before fitting this model to outcomes that are positively skewed. Missing scale item data will be handled as per questionnaire specific recommendations. Missing data handling methods aligned with the estimand of primary interest, such as multiple imputation, will be used to handle missing values. Pre-specified subgroup analyses irrespective of the findings in the primary outcome will be performed to explore heterogeneity in the treatment effect for the subgroups defined by centres and MND phenotype including a main effect for subgroup and the interaction between subgroup and treatment group in the model. The analysis models for all outcomes will adjust for the randomisation stratification variable centre as a main effect.

### Sample size

From the single-site RCT [16] and data from an additional 56 people with MND receiving usual care at Austin Health (Melbourne), it was estimated that the proportion adherent for usual care (control group) was 56% (95% confidence interval [CI] 41 to 70). It was estimated by expert consensus that a 20% absolute difference between the treatment arms is meaningful. With 41% (i.e. lower bound of the 95% CI) in the control group and 61% in the PSG group (ratio of ≈1.49), the total sample size is 194 (two-sided significance level of 5%, power of 80%). Accounting for a conservatively estimated drop-out rate of 20%, the total required sample size is 244 (122 per arm).

### Health economic evaluation

Cost-effectiveness analyses will be conducted alongside the trial from both a healthcare system and a patient/family perspective (including caregiver impacts). Additional extrapolation of the impact of NIV on survival will be incorporated. The model will be populated using outcomes data from the RCT and the subsequent cohort (NIV usage, health related quality of life (AQoL-8D) derived utility weights and survival). The costs of the intervention will be estimated from study protocols and budgets and the costs related to health resource use will be sourced from trial data, data linkage or self-report (hospitalisation, outpatient visits, disability supports and medication). A discount rate of 5% will be used for outcomes and costs incurred beyond 12 months. To access the effects of the uncertainty in estimates, a series of univariate sensitivity analysis will be conducted. Cost-effectiveness will be presented as a cost per additional hour of NIV use in the intervention group compared to the control group and cost per quality adjusted life year gained.

### Blinding

Participants, the clinical team and research staff collecting outcome measures will all be blinded to group allocation. The sleep scientist/registered nurse supervising the PSG will be unblinded to group allocation. The clinical team and others blinded to the treatment allocation will not access the PSG, ABG or other data. The reporting sleep physicians at each site who will provide a treatment recommendation (according to their usual practice) for both groups will input data on a form that will not indicate the treatment allocation.

### Trial management

Data will be inputted into a secure, password-protected, web-based database Adept (https://adeptrs.com/). Any paper records will be kept in a secure filing cabinet at each site. Data will be de-identified at source. Polysomnography will be performed, scored and reported locally. The PSGs will also be de-identified and transferred to the central research team electronically for central reporting to allow for sensitivity analysis of reporting methods between sites. This process has successfully been used in previous trials conducted by members of the research team [16].

The Trial Management Group (TMG), comprising the chief investigator, trial manager, implementation science lead and central trial staff are responsible for the day-to-day delivery and conduct of the trial and liaise directly with trial staff at each site. The TMG group meet weekly or more frequently if required and report directly to the Trial Steering Committee (TSC).

The TSC, comprising co-chairs from the community support organisation partners, chief investigator, site principal investigators, trial manager and implementation science lead, meet quarterly to review trial progress, review recommendations from the Data and Safety Monitoring Board (DSMB) and agree on action plans to address issues as they arise.

Site principal investigators are responsible for all aspects of the day to day running of the trial at their sites including identification of potential participants and consent for trial participation. Each site has a dedicated trial coordinator responsible for coordination of participant visits, data collection, data entry and liaising with the central team.

Comprehensive monitoring and auditing procedures will be conducted by a trial monitor who is independent of the site investigators and sponsor. The trial monitoring plan involves six-monthly database audits and annual site visits to each trial site to audit trial procedures, including protocol adherence and data quality. In addition to the site visits, each site will provide a quarterly progress report to the Steering Committee.

The DSMB, consisting of a respiratory physician, neurologist, MND Australia nominee and biostatistician, meet biannually (or sooner as required) throughout the trial to advise the Steering Committee. The DSMB monitor recruitment, withdrawals and safety. All adverse events will be collected, recorded and assessed by the DSMB. Adverse events that have a material impact on the conduct of the research will be reported to the reviewing HREC in accordance with the NHMRC Position Statement: *Monitoring and reporting of safety for clinical trials involving therapeutic products*, November 2016.

The results of the trial will be published in peer-reviewed journals and presented at conferences and scientific meetings internationally. Additionally, the trial outcomes will be provided to trial participants in a summary and the MND community through patient support organisations.

## Discussion

The results of this trial will demonstrate the effects of PSG-assisted titration of NIV to optimise NIV usage in people with MND. If a PSG can improve synchrony between the user and the machine, it is likely that NIV usage during the treatment period will be greater compared to the control group. Recent publications have confirmed that greater NIV use is associated with better survival in MND [3], making any and all strategies that successfully increase NIV adherence important aims of clinical care. Expert opinion and emerging evidence support the thesis that ‘better quality’ NIV provision in MND is an important pathway to better adherence [35, 36]. Additionally, NIV may be MND disease modifying per se through some or all of a reduction in chronic intermittent hypoxia or hypercapnia, relief from chronic sleep fragmentation or less respiratory event associated intracellular reperfusion stress [37].

Laboratory-based sleep studies are not used by all groups who treat respiratory failure in MND. If the current trial is successful, evidence of efficacy for PSG-assisted NIV commencement will present substantial implementation and practice change challenges for the field. To address this challenge, this trial has an embedded process evaluation which will be conducted contemporaneously with the 3TLA RCT. That protocol will be reported separately (Graco M, Berlowitz DJ, Sawyer A, et al: Polysomnographic titration of non-invasive ventilation in motor neurone disease (3TLA): protocol for a process evaluation of a clinical trial, forthcoming). Briefly, the mixed methods process evaluation aims to comprehensively evaluate the implementation of the 3TLA intervention in the trial sites, including barriers and enablers, and to assess the impact of implementation factors on trial outcomes and the mechanisms through which the 3TLA intervention produces change.

### Name and contact information for the trial sponsor

The University of Melbourne.

clinicaltrials-governance@unimelb.edu.au.

### Role of sponsor

The study sponsor and funders have no role in study design; collection, management, analysis and interpretation of data; writing of the report; and the decision to submit the report for publication, including whether they will have ultimate authority over any of these activities.

### Trial status

- ◦ Current protocol version 2.1 dated 21 February 2024
- ◦ Recruitment commenced 15 December 2021
- ◦ The trial was powered to cease recruitment when 244 participants were randomised, anticipated to be achieved by 28 February 2026. Trial commencement was delayed by 18 months due to the COVID-19 pandemic. These delays have significantly impacted recruitment rates and trial completion timelines. As of 20/08/2024, 43 participants have been recruited.

## Supplementary Information

Additional file 1. SPIRIT checklist for Trials.

Additional file 2. SPIRIT figure.

## Data Availability

In accordance with recommendations of the International Committee of Journal Editors, individual participant data will be retained and shared. Pseudonymised data will be available with no end date to selected trial researchers along with a separate password-protected dataset linking trial identifiers to trial participants. Anonymised individual participant data that underlie the results reported in publications will be stored in a secure online repository, beginning 9 months following publication with no end date [11, 38].

## Abbreviations

ABG: Arterial blood gas
ALS: Amyotrophic lateral sclerosis
AQoL-8D: Assessment of Quality of Life
cmH_2_O: Centimetre of water
CO_2_: Carbon dioxide
DSMB: Data and Safety Monitoring Board
EPAP: Expiratory positive airway pressure
FEV1: Forced expiratory volume in 1 s
FVC: Forced vital capacity
IPAP: Inspiratory positive airway pressure
MEP: Maximal expiratory pressure
MIP: Maximal inspiratory pressure
mmHg: Millimetre of mercury
MND: Motor neurone disease
NIV: Non-invasive ventilation
PROMs: Patient-reported outcome measures
PSG: Polysomnography
PtcCO_2_: Transcutaneous carbon dioxide
RCT: Randomised controlled trial
REM: Rapid eye movement
SNIP: Sniff nasal inspiratory pressure
