# Supplementary material for: Polysomnographic titration of non-invasive ventilation in motor neurone disease (3TLA): study protocol for a randomised controlled trial
Source: Trials. 2025 Jan 6;26:10. doi: 10.1186/s13063-024-08464-4 (PMC11705826; doi:10.1186/s13063-024-08464-4)
Supplement: Supplementary file 2 — Additional file 2. SPIRIT figure. [file 13063_2024_8464_MOESM2_ESM.docx]

|  | **STUDY PERIOD** | | | | | | | |
| --- | --- | --- | --- | --- | --- | --- | --- | --- |
| **PERIOD** | **Enrolment** | **Daytime NIV initiation** | **Acclimatisation** | **PSG1 & Allocation** | **Intervention** | **PSG2** | **Cohort follow-up** | |
| **TIMEPOINT** | **Week -1** | **Day 0** | **Day 0–Day 20** | **Night 21** | **Day 22–Day 69** | **Night 70** | **26-week** | **52-week** |
| **VISIT** |  | **1** |  | **2** |  | **3** |  |  |
| **ENROLMENT:** |  |  |  |  |  |  |  |  |
| Eligibility screen | x |  |  |  |  |  |  |  |
| Informed consent |  | x |  |  |  |  |  |  |
| Daytime NIV set-up |  | x |  |  |  |  |  |  |
| **INTERVENTION:** |  |  |  |  |  |  |  |  |
| Randomisation |  |  |  | x |  |  |  |  |
| NIV titration PSG or Sham PSG |  |  |  | x |  |  |  |  |
| **ASSESSMENTS:** |  |  |  |  |  |  |  |  |
| Repeat PSG* |  |  |  |  |  | x |  |  |
| Demographics |  | x |  |  |  |  |  |  |
| Medical history |  | x |  |  |  |  |  |  |
| MND Medications |  | x |  |  |  |  |  |  |
| NIV use |  |  | x |  | x |  | x | x |
| Clinical contact |  |  | x |  | x |  |  |  |
| Respiratory function |  | x |  | x |  | x | x | x |
| Physiological and objective sleep quality |  |  |  | x |  | x |  |  |
| Gas exchange |  |  |  | x |  | x |  |  |
| Patient reported outcome measures |  | x |  |  |  | x | x | x |
| Caregiver burden |  | x |  |  |  | x | x | x |
| Healthcare utilisation |  | x |  |  |  | x | x | x |

Abbreviations: MND, motor neurone disease; NIV, non-invasive ventilation; PSG, polysomnography.

* No settings will be changed during the repeat PSG for either the Intervention or Control groups.
